# Trend and Predictors of Breastmilk Feeding among Very-Low-Birth-Weight Infants in NICU and at Discharge

**DOI:** 10.3390/nu15153314

**Published:** 2023-07-26

**Authors:** Pasqua Anna Quitadamo, Federica Zambianco, Giuseppina Palumbo, Massimiliano Copetti, Maria Assunta Gentile, Antonio Mondelli

**Affiliations:** 1 Neonatal Intensive Care Unit, Casa Sollievo della Sofferenza, 71013 San Giovanni Rotondo, Italy; palumbogiuseppina@tiscali.it (G.P.); ma.gentile@operapadrepio.it (M.A.G.); a.mondelli@operapadrepio.it (A.M.); 2 HMB, Casa Sollievo della Sofferenza, 71013 San Giovanni Rotondo, Italy; 3 San Raffaele Faculty of Medicine, University of San Raffaele Vita-Salute, 20132 Milano, Italy; federicazambii@gmail.com; 4 Statistical Department, Casa Sollievo della Sofferenza, 71013 San Giovanni Rotondo, Italy; m.copetti@operapadrepio.it

**Keywords:** mothers’ own milk, preterm feeding, VLBW

## Abstract

Mothers’ own milk (MOM) for premature babies is considered a life-saving drug for its proven protective action against the complications of prematurity and for effects on outcome in the short and long term, especially neurological ones. We studied the use of MOM for infants weighing <1500 g for a period of 5 years, evaluating the trend over time and the impact of some variables on human milk feeding performance. Statistical comparisons concerned the rate of feeding with breast milk during a stay in an NICU and at discharge with respect to two types of variables: (1) maternal and neonatal characteristics (gestational age, birth weight, type of pregnancy (whether single or twin), maternal age) and (2) feeding characteristics (time of the start of minimal enteral feeding and availability of MOM, days until the achievement of full enteral feeding). Group comparisons were performed using ANOVA or *t*-test for continuous variables and Pearson chi-squared test or Fisher exact test for categorical variables. We observed an increase, between 2017 and 2021, in MOM use (*p* = 0.003). The availability of the own mothers’ milk occurred, on average, on the fourth day of life and improved over the years. The start of minimal enteral feeding (MEF) with human milk averaged 1.78 days, and 54.3% of VLBWs received MEF with donor milk on the first day of life. The average percentage of feeding with the mothers’ milk at discharge was 47.6%, with 36.1% of exclusive MOM and an increase from 45.8% in 2017 (33.3% exclusive) to 58.82% (41.18% exclusive) in 2021. The mean average daily growth of the weight improved (*p* < 0.001) during this period, and there was no statistical difference between infants fed with maternal milk and those fed with bank milk. Older maternal age, early-start feeding with maternal milk and low gestational age had a statistically significant impact on feeding with MOM at discharge.

## 1. Introduction

Preterm birth is the leading cause of death for those under five years of age, responsible for about one million deaths in 2015 [1,2,3].

The international average mortality rate for infants weighing less than 1500 g is 14.6%, according to data from the Vermont Oxford Network. Italy is among the countries with the lowest mortality rate, with an average percentage of 11.9% (Network InnSin).

There is wide variation in preterm birth trends in European countries [4]. Preterm and low-birth-weight infants are among the most vulnerable in our society [5] and deserve special attention as well as a greater commitment to care in all aspects, especially nutritional ones, which impact mortality and morbidity. MOM for premature babies is considered an important opportunity for life and health because it reduces the risk of major complications of prematurity but also an investment for life for its long-term outcome effects, including neurological and cognitive ones [6,7,8]. Much has changed over the years, given the awareness that correct nutritional practice in the early periods of life can affect present and future well-being [7,8]. Scientific evidence regarding the benefits of human milk is recognized by the world’s leading health authorities and international bodies [9,10,11]. 

In fact, even politics, both nationally and internationally, has given and is giving important contributions to the promotion of breastfeeding, with official statements, specific dedicated documents, guidelines and reports. So, small signs of a greater diffusion of breast milk use in NICUs are recorded, which is a recognized index of quality. The exception is Sweden [12], one of the most historically virtuous countries regarding breastfeeding, where the percentage of extremely low-birth-weight (ELBW) babies fed at discharge with MOM decreased from 55% in 2004 to 16% in 2016. 

Data regarding the type of feeding at discharge are more present in the literature, but those concerning the first weeks of life in the NICU, crucial for the development of very-low-birth-weight (VLBW) infants, are sparse. We refer to the nutrition of the MOM obtained via expression of the breast and then to that directly from the breast.

In this study, in addition to estimating MOM usage rates, we evaluated the three key moments of nutritional care in NICUs: the start of enteral feeding, the achievement of full enteral feeding (FEF) and the type of feeding at discharge. These are key elements for assessing the quality of care. Another aspect of interest was the identification of factors that could favor the use of breast milk in the NICU and at home.

The study arose from the feeling that, despite all of the efforts to guarantee human milk to all premature babies born in our territory (thanks to the availability of donated milk from the bank), the gold objective to provide VLBWs and ELBWs mainly with their mother’s milk has not yet been fully achieved. The analysis also stems from the awareness of the need for monitoring this important aspect, which is necessary to be able to plan proactive actions. Improvements can arise, for example, by acting on these variables that could be facilitators. Since the literature suggests that the start of feeding (minimal enteral feeding) and the first availability of the mother’s milk are the elements most often indicated as predictors of breastfeeding at discharge and in the following months, we chose them for our analysis. We added the study of possible associations with maternal or neonatal factors that could guide us regarding the identification of categories of mothers or newborns that may require more attention. As a secondary aim, we wanted to evaluate the growth trend in VLBWs fed with human milk, another important aspect for the outcome of these infants, also comparing the population of premature babies who received bank milk and those who received breast milk, another topic of interest and discussion.

## 2. Description of the Study

### 2.1. Objectives

We aimed to *evaluate* the use of MOM in NICU and at discharge: n° VLBWs fed with MOM in the first weeks of life, n° VLBW discharged with MOM, the trend over the years and a correlation between maternal and neonatal factors with MOM feeding or not in the NICU and at discharge; *analyze* the start times of minimal enteral feeding and of the availability of MOM, the timing of reaching full enteral feeding and the correlation of these factors with the frequency of feeding with MOM in the NICU and at discharge. 

The last aim is to identify the margins for an improvement in the nutritional performance of MOM, with actions aimed at the infants most at risk of not receiving their mother’s milk.

### 2.2. Design, Setting and Methods

We obtained informed consent from the mothers via a dedicated and signed form and the approval of the hospital’s ethics committee.

The sample included VLBWs admitted to the NICU from 2017 to 2021. A total of 97 VLBWs were recruited. Transferred infants, babies with malformations with intrinsic interference with nutrition and those born to mothers with pathologies incompatible with milk production and extraction were excluded. 

The data were extrapolated from medical records, care cards and Human Milk Bank’s database. The data collected were: neonatal and maternal data: gestational age, birth weight, type of pregnancy (whether single or twin) and maternal age; feeding data: timing of: the start of the Minimal Enteral Feeding, the availability of the maternal milk and the achievement of the Full Enteral Feeding; the type of nutrition at discharge and data relating to the length of stay and weight increase. Nutrition with MOM during hospitalization means a minimum of 2 continuous weeks with exclusive or prevalent MOM (MOM > 50%). Nutrition in NICU and at discharge is the parameter of study. Neonatal, maternal and feeding data were used as variables. Statistical comparisons concerned data on feeding with MOM in the NICU and at discharge with respect to variables. 

Specifically, two types of comparison were conducted: in a test, the group of VLBWs that received breast milk vs. the group of VLBWs that did not receive breast milk (but only bank milk); in a second test, 3 populations were correlated in two comparisons: 1. VLBWs discharged only with FM (Formulated Milk) vs. VLBWs discharged with MOM and vs. VLBWs discharged with MOM + FM and 2. VLBWs discharged with FM vs. VLBWs discharged with MOM. 

Demographical and clinical characteristics were reported as mean and standard deviation for continuous variables and as frequency and percentage for categorical variables.

Group comparisons were performed using ANOVA or t-test for continuous variables and Pearson chi-squared test or Fisher exact test for categorical variables. A *p*-value <0.05 was considered statistically significant. All analyses were performed using the software R-project (version 4.2.0).

## 3. Results

The characteristics of the sample of the 97 VLBWs recruited are summarized in Table 1, while the trend over the years is represented in Table 2.

The average maternal age was 32.62 years, but it grew over the years from 31.86 years in 2017 to 35.65 years in 2021.

Further, 69.5% of mothers of premature babies provided their own milk. We observed an increase, between the year 2017 and 2021, of MOM users (*p* = 0.003), except for 2020, where the low percentage of VLBWs fed with MOM is probably due to the small number of observations in this year. In particular, the percentage of feeding VLBWs with mother’s milk has grown over the years in a statistically significant way, with a positive peak of over 91% in 2019 and a negative trend of 14.9% in 2020, the year of the COVID-19 pandemic. 

The availability of the mother’s milk occurred, on average, on the fourth day of life; these data improved over the years, settling at the third in 2021. The start of Minimal Enteral Feeding with human milk averaged 1.78 days, and 54.3% of VLBWs received MEF with Donated Milk (DM) on the first day of life (50% within the first 6 h) and 36.9% on the second day, while MEF with maternal milk started on day 2 for 10%, on day 3 for 21.6% and between the fourth and fifth day for 51.6%. 

**Figure 1:**
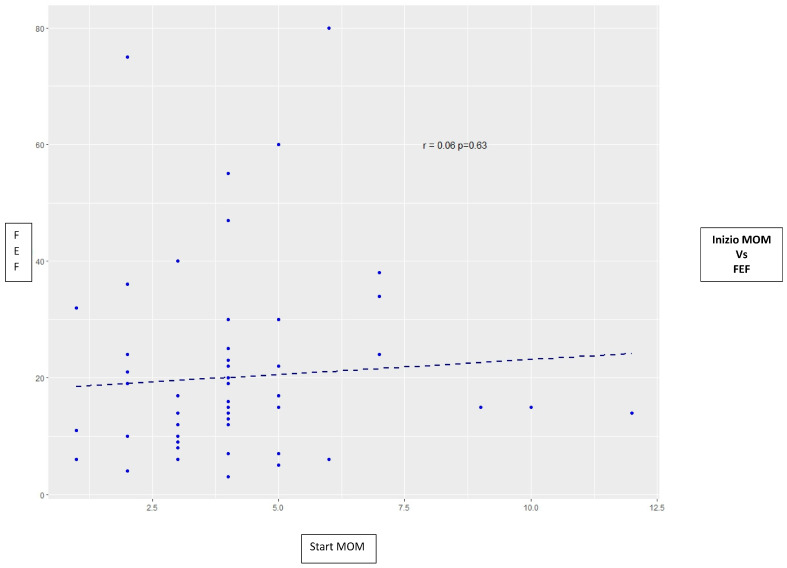
Scatterplot (points) and correlation (regression line) between the time starting MOM (Mother Own Milk) in days and the time of reaching Full Enteral Feeding (FEF) in days. We observed a lack of correlation (r = 0.06, *p* = 0.63) between the time starting MOM and the time of reaching FEF. These two variables don’t affect each other. This element could be attributed to the fact that more than the times, is the type of feeding that affects the timing of reaching Full Enteral Feeding as described in the literature [13,14].

On an average stay of 62 days, the achievement of the Full Enteral Feeding occurred in 18 days, and a median of 14. 26 (26.8%) preterm babies reached the FEF within the tenth day of life. The trend of the FEF compared to the start of the MOM availability is shown in Figure 1.

The average MOM feeding at discharge was 47.6%, with 36.1% exclusive MOM (EMOM), with an increase from 45.8% in 2017 (EMOM 33.3%) to 58.82% (EMOM 41.18%) in 2021.

The average daily weight gain was 24 g, with an increase over the years from 21.08 g in 2017 to 31.36 g in 2021. Moreover, we also observed growth in the mean weight increase (*p* < 0.001) during this period. We found no significant difference between infants fed with their mother’s milk and those fed with bank milk. 

There was no statistical significance of gestational age, birth weight, type of delivery and pregnancy (whether single or twin) and maternal age on the availability of MOM during hospitalization (Table 3).

The maternal age and the onset of feeding with breast milk have a statistically significant impact on the type of feeding at discharge (Table 4a,b) if the three categories (Exclusive MOM, Mixed MOM, FM) are distinguished, and if two groups, MOM (Exclusive MOM + Mixed MOM) and FM are compared, gestational age at birth is also significant.

All patients discharged with breast milk alone or breast milk with FM started with MOM feeding earlier than those discharged with preterm formula (*p* = 0.032). 

## 4. Discussion

Preterm infants are a high-risk population, and prematurity is a leading cause of neonatal morbidity and mortality. Feeding with breast milk during the first days and months is one of the most impactful factors on the health of these vulnerable infants, as it reduces the incidence and severity of complications associated with prematurity and their related costs [15,16,17]. In addition, exposure to MOM, particularly in the first weeks of life, also improves long-term outcomes, especially the neurodevelopment [18,19,20], and reduces disease and rehospitalization rates in the first year of life [17]. These effects are attributable to immunological, antimicrobial, anti-inflammatory, antioxidant, epigenetic, growth-promoting and intestinal colonizing functions exerted by multiple bioactive factors, many of which are present in higher concentrations in the breast milk of mothers of preterm infants [6]. The protective action is a dose–response relationship, with higher and prolonged doses of MOM providing maximum protection [21]. Breast milk exposure rates vary widely between studies.

The first discussion item is just the fragmentation and inhomogeneity of the data on this topic, which instead is fundamental in the care of infants in NICU, because it conditions their survival, their outcome and, therefore, their future. In the literature, there are monocentric reports like this and rare and unsystematic multicenter reports or data from national or international registers. Institutions dealing with child health should plan a program of constant monitoring of this aspect of life in NICU, which starts from the individual realities and extends to nations and continents.

In addition, the analysis of each center and the sharing of data have value for improving quality, whose tools cannot disregard from the collection, analysis, understanding and communication of data.

The percentages of feeding with MOM of 69.5% recorded in our NICU are not satisfactory but better than the European [22,23,24,25,26] (France 49%, Germany 47–60%, 44% in Portugal, 53.6–78% in Greece, 49% in Sweden) and Chinese [27] data (58%) and in line with those of the United States [28,29,30] (70–75%); our values have improved over the years, except for 2020, the year of the COVID-19 pandemic. This trend is common to other countries that recorded a 10–20% increase over the years [22]. In contrast is the resounding data from Sweden [12], which decreased from 55% of exclusive feeding with MOM of very preterm babies in 2004 to 16% in 2013, from 41% to 34% in preterm newborns between 28 and 31 weeks and from 64% to 49% in moderately preterm infants (GA 32–36 weeks). 

The most virtuous model remains Brazil, which has a national standardized integrated system of assistance in NICU and promotion of breastfeeding and donation, which it also exports to other states. In Brazil [31], the prevalence of exclusive breastfeeding was 65.2% at discharge, 51% at 3 months and 20.6% at 6 months. 

Our data confirm the devastating effect of the pandemic on feeding babies with MOM (Table 2), as well as the dramatic reduction in milk donation. In 2020, the phenomenon was so important that all associations and scientific societies drew up documents to try to remove fears, reassure the public about breastfeeding and revive the spirit of generosity of women who have a surplus of milk. In contrast with the general trend, in our HMB [32], milk donations grew in those critical months. Most probably, this result was due to the spirit of solidarity, which was very strong in the first COVID-19 period, together with the sharing of our HMB with women donors, which has never stopped and actually strengthened during those difficult days. 

Initiation of MEF with Donated Milk averaged 1.78 days and, with MOM, 4.11 days. In our study, the start of MEF with MOM has seen improvements over the years, from an average of 4.44 days to 3.3 days. The start of feeding with MOM was a factor that significantly influenced the type of milk at discharge (*p* = 0.009), in line with other authors [30], who found that the main predictor of breastfeeding at discharge was the reception of MOM by the third day of age. It is known that the first hours and days after birth are a decisive moment for the start of breastfeeding. 

Although there is no clear consensus in the guidelines, more reports recommend early and progressive enteral feeding [33,34,35,36,37,38,39,40,41]. In particular, it is advisable to start in the first 6 h [42], if the clinical conditions allow it, and, in any case, within the first 24–72 h of life. Initiation of enteral feeding within 72 h of birth [43,44] appears to reduce mortality, risk of sepsis, of bronchodysplasia and length of hospital stay. To have maternal colostrum readily available, it is important to avoid a delay in secretory activation, also because the transition from differentiation to secretory activation within 72 h of birth has an impact on long-term milk production [45]. Early, frequent and effective expression is crucial for both the effect on health and on the duration of breastfeeding.

It is believed that for premature babies, the early expression of breast milk has a value comparable to the early onset of breastfeeding for full-term infants on the success of prolonged exclusive breastfeeding. Parker et al. [46]. reported that first milk expression within 8 h was higher than 9–24 h with respect to maximal duration of provision of mother’s milk for hospitalized VLBW infants but emphasized that to establish a causal relationship between timing of first milk expression and long-term lactation success, randomized control trials are needed.

This suggests intensifying compliance with the breast stimulation protocol that recommends starting within 6 h of delivery [45]. In this study, data on the time of the first breast stimulation are missing. However, data in the literature show unsatisfactory percentages for mothers who start expressing milk within 6 h of delivery (36% in Finland [47], 17% in Japan [48], 3.3% in India [49]).

The most effective intervention to achieve the objective of an early and frequent expression of milk is preventive information. When mothers receive adequate evidence, with scientific and practical content, about the importance of their breast milk, the results are more satisfactory [50].

In our maternity unit, all women after premature birth are equipped with a breast pump, along with indications and recommendations on the practice of systematic breast stimulation; nevertheless, we would like to emphasize that the care of mothers in this aspect, ranging from information to systematic dialogue, monitoring of milk production and support for extraction and direct breastfeeding, must become central in the day-to-day economy of assistance for premature babies. 

In our study, one of the most important elements for the aim of ensuring a longer duration of exposure of VLBWs to breast milk, the transition to the breast, was not analyzed. NICU infants face a unique set of challenges, and infants’ progression to breastfeeding is often complicated by clinical criticalities, gastro-immaturity and underlying medical comorbidities. Supportive practices, such as oral therapy, skin-to-skin care and non-nourishing sucking, are of great importance for the earlier initiation of breastfeeding but also for the development and relationship of the dyad. Research [51,52,53] has shown that these practices support breast milk volumes and the baby’s transition from enteral feed to breastfeeding, thus leading to higher breastfeeding rates. These are carried out systematically in our NICU, and this could explain the improvement in data over the years, but they must be better accompanied by a total cultural change of pace in the monitoring of and continuous improvements in care.

Maximum protection induced by breast milk is achieved when vulnerable infants receive high doses and long exposure to MOM [33]. Daily volumes of at least 500 mL before day 14 are indicated to be associated with significantly higher breastfeeding rates at discharge [54]. Breast milk volumes should be monitored to adapt clinical practice interventions. There are sporadic reports on this focus. One of the few examples is the mPINC survey, a biennial census of all maternity care hospitals in the United States and territories to monitor practices and policies related to infant feeding.

The achievement of FEF occurred within 14 days for 65% of VLBWs, with a median of 15 days. These are also important data to monitor, because the achievement of FEF translates into the suspension of parenteral nutrition and central venous access, with all that this entails in terms of complications related to both factors. We did not find comparable data on the average time to reach the FEF in the literature, and this element could also be a starting point for dedicated monitoring [55,56]. Two recent systematic reviews and meta-analyses [57] and other studies [13] show that the use of HM (MOM or DM) vs. the formula leads to a better food tolerance, allows for the start of enteral feeding earlier, to increase milk volumes more rapidly with the faster achievement of FEF and allows for reducing the use of parenteral nutrition and the related risks. Our NICU with attached HMB can be an example [14]. 

In this regard, it should not be surprising that, in our study, no association was found between the use of MOM and the achievement of FEF or the length of hospitalization, which also reduced over the years, because no FM was used but only human milk since 2010.

In other NICUs, central catheters are removed when patients achieve an EF of 100 mL/kg/day [58]. 

Even without a precise rule, but with an individualized approach, our cutoff varies between 80 and 100 mL/kg/day, and we have a dedicated protocol on the progression of enteral feeding. There are some studies that have compared the effects between a slow increase in intake and a more aggressive progression of volumes.

The evidence-oriented literature is more likely to consider that enteral feeding, specifically early-onset and faster enteral advancement, impacts on preterm infants’ health during the first month of life, acting on the intestine, promoting its maturation and a more beneficial microbiome composition but also reducing inflammation and improving brain growth and neurodevelopment. Instead, delaying MEF and FEF may decrease the functional adaptation of the gastrointestinal tract and disrupt microbial colonization patterns [59,60] and promote inflammation [19] that increases the risk of comorbidities [61,62], and therapies to manage them, like steroid use, can impair linear growth [63]. Small and large randomized trials [33,40] seem to show that rapid enteral advancement and even early aggressive feeding regimens are feasible in very small infants (750–1250 g), because they are not associated with increased risk of feed intolerance or NEC. Maybe they do not significantly reduce mortality or morbidity during hospitalization but decrease the days to reach FEF and reduce the mean NICU stay duration. Also, in a review in 2019 [57], a more sustained advancement appeared to be safe and feasible in stable VLBW infants with birth weight > 1000–1200 g, although it is believed that a large, randomized trial is needed to confirm the benefits. 

In addition, the management of the advancement of enteral inputs also changes with respect to the country, since, in many high-income countries, the conservative approach with a slower increase in volumes prevails, fearing that early FEF could increase the risk of hypoglycemia, food intolerance, gastro-esophageal reflux, ab ingestis and NEC in very preterm infants or VLBW [40,41]. However, in low- and middle-income countries with fewer resources for neonatal care, the practice tends to favor the early introduction and advancement of enteral feeds for stable infants [64].

Others [57] indicate that slow advancement of enteral feed volumes compared to faster rates probably does not reduce the risk of NEC, death or food intolerance in very preterm or VLBW infants, and, instead, may slightly increase the risk of invasive infection. 

We also consider once again that the increase in the duration of parenteral nutrition is associated with infectious and metabolic complications that increase mortality and morbidity, prolong hospital stay and negatively affect growth and development [65]. For these reasons, some authors [22] believe that an early transition to full-volume enteral feeding should be seen as an ideal therapy to promote appropriate growth, body composition and development in preterm infants. 

Breastfeeding at discharge is a more studied topic. The data are very unsatisfactory, with an average percentage of BF of 47.4%, if we consider the objectives indicated by national and transnational institutions. The numbers have grown over the years, except for 2020, the year of the pandemic. Data are in line with those reported by VON [66], where National data from more than 800 NICUs showed that the provision of human milk at discharge among VLBW infants increased from 44% in 2008 to 52% in 2017.

In Germany, 60.1% of patients were discharged with exclusive MOM feeds out of a sample of 368 premature babies [23]. This rate was higher than in the EPIPAGE-2 cohort study [22], which reported 25% exclusive feeding and 47% of some MOM feeds at discharge in children under 32 weeks. In Greece, 48% of breastfeeding at discharge is reported [26]. 

In a cohort from 11 countries in 19 European regions, 58.5% of preterm infants < 32 weeks received human milk at discharge [22], with important regional differences in breastfeeding rates and significant variations ranging from 36% to 80%. Rates ranging from 49% to 87% among NICUs have been reported in the United States [30]. 

In a multicentric study [67], 45% of infants < 1500 g birth weight and 23% of infants > 2500 g did not receive MOM at discharge in Italy. In a more recent report [68] of a single NICU, 66% of preterm infants received any breastfeeding at discharge, of which 27% were exclusively breastfed. A more up-to-date Italian survey would be desirable.

Very few reports have evaluated breastfeeding in the months following discharge.

A few Portuguese studies [69] have reported a low and variable (1.0% to 27.0%) prevalence of exclusive breastfeeding at 6 months and any breast milk at 12 months (8.0% to 12.0%). In another Greek study [70], 58.1% were exclusively breastfed during the first month, with a gradual decrease to 36.9% to the third month of life and 19.4% to the sixth. The prevalence of breastfed infants reached 14.7% and 7.5% at the ages of twelve and eighteen months, respectively. 

Young maternal age is indicated in some studies as a risk factor for NMOM (No Mother’s Own Milk) feeding at discharge. Every year of maternal age was associated with a 1.24-fold increase in direct breastfeeding at hospital discharge, or infants with mothers younger than 25 years were 30% less likely to be breastfed than infants with older mothers [71]. Mothers aged <25 years ceased breastfeeding more often before discharge and before six months of age than mothers over the age of 25 [72].

In our study, feeding with HM at discharge was 54.5% for mothers aged 36 to 40 years and 21.4% for those aged 21 to 30 years with a statistically significant difference (*p* value < 0.005). Maternal age was a factor that significantly influenced breastfeeding at discharge. Thus, younger mothers represent a category to be supported more.

Of the 13 gestational age ≤ 25-week infants, 77% received MOM in the first few weeks and 61.5% received at discharge. For the 30 infants born GA ≥ 30 weeks, 58% received MOM in NICU and 35% at discharge. A statistically significant association was found between gestational age and MOM feeding at discharge. In our previous report [73], mothers of preterm babies of GA ≤ 29 weeks were more likely to produce breast milk.

Probably, the care dedicated to the mothers of VLBW is more effective, both for the predisposition of mothers who recognize, in the extraction of milk, the only act they can make available for the survival and health of their children, and for the health personnel, who welcome a newborn who will undergo a long hospitalization. The data are interesting, considering that the milk produced by the mothers of VLBWs has a specific composition tailored to this fragile category of premature babies [73,74]. But, it is objectively paradoxical that babies born at a higher gestational age and who are, therefore, more able have been fed less with MOM.

In our population, 37.5% of VLBW premature babies were twins and, of these, 50% received MOM in NICU, compared to 66.7% of those born from single pregnancies and with unexpectedly higher rates of MOM at discharge (48.3% for twins vs. 43.1% for singles). This confirms a trend in our NICU [75], and comparing it with the few other reports available, we can state that multiples were not at higher risk of NMOM feed than singletons in some cases. But, the data on multiple births are controversial, since some studies show an association with exclusive breastfeeding [76], while others [71,77,78] show an association with NMOM feeds or discontinuation of breastfeeding before six months of age. Further studies to clarify this point would be needed.

The type of delivery, though with clearly prevalent CS, did not affect the type of feeding, either during hospitalization or at discharge. 

Weight growth is one of the most important factors in the management of VLBWs for their implications on outcomes. Average daily weight gain improved significantly, and this is an encouraging achieved goal.

There is a debate about the comparison between breast milk and Donated Milk and their impact on VLBW growth. In our report, there is no statistical significance in monitoring weight gain with respect to the type of diet (in our NICU, only human milk is used for VLBW); namely, there was no significant difference in the daily weight trend between those fed with DM and those fed with their mother’s milk, both fortified at the right time. Santiago [79] et al. conducted a review with heterogeneous results regarding weight gain and linear growth in infants fed with human milk, fortified human milk or preterm formula [72]. The data in the literature are controversial, but it was shown that the presence of HMBs and/or the use of DM in NICU are associated with an increased incidence of breastfeeding, both during hospitalization and at discharge [80]. This is also our experience.

An international survey [81] on differences in feeding practices found that most NICUs with access to DM started enteral feeding earlier and progressed more rapidly. Units without access to DM often delayed the introduction of enteral feeds until MOM was available [82].

We believe that providing DM to vulnerable infants who do not have MOM can save lives and raise awareness of the value of breastfeeding and human milk in NICU and in the community. 

A few modifiable factors were included in our study. However, it should be remembered that in the NICU, the most significant results are obtained when the staff is trained to promote breastfeeding and donation [83]. The role of health professionals in a multidisciplinary framework is fundamental in supporting mothers in breastfeeding in neonatal intensive care, in accompanying early and frequent expression of milk, in promoting skin-to-skin care and breastfeeding when conditions allow it. They should be prepared to identify and counteract psychological, physical, social and cultural barriers to successful milk extraction and to breastfeeding. It is a question of priorities that should be established and integrated into the cultural background and in daily actions.

**What this study adds**

The novelty elements of the study are listed in Box 1.

Box 1 **What’s new in this study.**

  Rates of feeding with MOM during hospitalization and at discharge follow a trend common to other countries, increasing by 13% in recent years, and this is encouraging although still not satisfactory.

  We can indicate the early start of Minimal Enteral Feeding with MOM as the main predictive element of a greater feeding of VLBW with breast milk (quantity and dura-tion), and this suggests that all the most effective methods to obtain the availability of expressed breast milk should be utilized as soon as possible.

  The mothers of premature babies weighing <1000 g provided more milk, and this element sends the message that milk can be produced even at very low gestational ag-es.

  Younger women breastfeed less and, therefore, need to be followed more.

  There is no significant gap in weight trends between VLBWs fed with their moth-er’s milk and those with donated milk, removing one of the main fears related to the use of bank milk.

  This analysis should be conducted systematically in our and every NICU, because only through monitoring can we improve this crucial aspect of care for a very im-portant category of newborns.

## 5. Conclusions and Relevance

VLBWs pose a significant nutritional challenge. Feeding rates with MOM during hospitalization and at discharge have improved, increasing by circa 13% in recent years, a common trend to other countries, but these levels are still very far from those indicated by the WHO and the national and international bodies and agencies. The year of the pandemic was devastating for our NICU, with a marked reduction in the use of MOM and in breastfeeding. The timing of the start of EF with MOM, which, over the years, is back to 72 h of life, was found to be the most important predictive element of volume and duration of exposition to MOM. ELBW infants’ mothers extract more milk, and young mothers feed their preterm babies with MOM less. No significant differences were registered in the trend of the VLBW babies’ weight between those fed with MOM and those fed with DM. The literature lacks systematic and coherent data, which are useful for a necessary monitoring of feeding in NICU and in the months after discharge, if you consider the quantitative and qualitative impact of the use of mothers’ milk on development and outcome and, therefore, on the future of this vulnerable category of children. 

## 6. Strengths and Limitations

The study of nutrition in NICU and at discharge, scanning the data of times and percentages, so as to give a picture of the food performance of VLBWs over a fairly long period, is an important element from our point of view to propose and share. Recall that it is one of the quality indices of care in NICU, recognized globally.

The limitations are represented by the fact that it is a monocenter study, and multicenter studies are desirable, and by the absence of monitoring data on the transition to direct breastfeeding and the analysis of modifiable factors (care in NICU, caregiver competences, NICU open to parents, etc.) that could become the subject of subsequent studies.

## Figures and Tables

**Table 1:** Numerical data of the sample expressed as Mean (SD), Median (Q1, Q3), Min–Max.

	(*n* = 97)
Gestational Age	
Mean (SD)	28.56 (2.73)
Median (Q1, Q3)	29.00 (27.00, 30.00)
Min–Max	23.00–35.00
Birth Weight	
Mean (SD)	1091.48 (287.93)
Median (Q1, Q3)	1110.00 (870.00, 1320.00)
Min–Max	520.00–1495.00
Year	
2017	24 (24.7%)
2018	24 (24.7%)
2019	24 (24.7%)
2020	8 (8.2%)
2021	17 (17.5%)
Single/Twin	
Twin	36 (37.5%)
Single	60 (62.5%)
Delivery	
Vaginal	14 (14.6%)
Cesarean Section	82 (85.4%)
Maternal Age	
Mean (SD)	32.62 (6.11)
Median (Q1, Q3)	33.00 (28.50, 37.50)
Min–Max	19.00–46.00
Donated Milk mL	
Mean (SD)	4015.88 (3840.05)
Median (Q1, Q3)	2900.00 (500.00, 7100.00)
Min–Max	12.00–12,200.00
Start Minimal Enteral Feeding	
Mean (SD)	1.78 (1.03)
Median (Q1, Q3)	2.00 (1.00, 2.00)
Min–Max	1.00–7.00
Maternal Own Milk	
NO	29 (30.5%)
YES	66 (69.5%)
Start Maternal Own Milk	
Mean (SD)	4.11 (1.93)
Median (Q1, Q3)	4.00 (3.00, 5.00)
Min–Max	1.00–12.00
Full Enteral Feeding	
Mean (SD)	18.53 (14.30)
Median (Q1, Q3)	15.00 (9.00, 22.00)
Min–Max	3.00–80.00
Days of hospitalization	
Mean (SD)	62.14 (25.76)
Median (Q1, Q3)	58.00 (45.25, 76.00)
Min–Max	22.00–161.00
Weight at discharge	
Mean (SD)	2538.55 (529.59)
Median (Q1, Q3)	2390.00 (2157.50, 2802.50)
Min–Max	1781.00–4960.00
Feeding at discharge	
Formulated Milk	51 (52.6%)
Maternal Own Milk	35 (36.1%)
Maternal Own Milk + Formulated Milk	11 (11.3%)
Average daily weight increment	
Mean (SD)	24.02 (7.22)
Median (Q1, Q3)	23.00 (19.67, 26.85)
Min–Max	12.20–71.30

**Table 2:** Table of trends in the years of neonatal, maternal and nutritional variables.

Year	2017	2018	2019	2020	2021	*p*
n°	24	24	24	8	17	
GA (mean (SD))	27.29 (2.37)	28.58 (2.89)	29.00 (2.59)	29.25 (3.28)	29.35 (2.57)	0.097
Weight (mean (SD))	1030.00 (316.32)	1085.46 (281.31)	1139.29 (298.99)	1052.50 (243.35)	1137.65 (270.64)	0.680
Single (%)	17 (70.83)	16 (66.67)	15 (65.22)	5 (62.50)	7 (41.18)	0.372
CS (%)	18 (78.26)	21 (87.50)	18 (75.00)	8 (100.00)	17 (100.00)	0.117
Maternal age (mean (SD))	31.86 (6.15)	32.38 (6.98)	31.04 (5.71)	33.75 (4.13)	35.65 (5.48)	0.168
MOM = YES (%)	16 (66.67)	15 (62.50)	21 (91.30)	1 (14.29)	13 (76.47)	0.003
Start MOM (mean (SD))	4.44 (2.16)	4.47 (2.53)	4.00 (1.63)	4.00 (0.00)	3.30 (1.16)	0.602
DM mL (mean (SD))	3211.25 (3324.12)	4599.29 (4045.35)	3200.57 (3510.58)	9883.33 (2116.99)	2125.00 (1935.94)	<0.001
Start MEF (mean (SD))	1.52 (0.73)	2.04 (1.40)	1.67 (0.48)	2.38 (1.92)	1.65 (0.61)	0.180
FEF Achievement (mean (SD))	22.77 (21.76)	15.58 (7.37)	16.22 (10.72)	20.88 (14.95)	19.89 (12.75)	0.433
Length of stay (mean (SD))	65.71 (30.32)	68.54 (28.76)	60.58 (22.10)	59.50 (19.34)	52.12 (21.49)	0.331
Average daily weight increment (mean (SD))	21.08 (3.44)	22.27 (4.46)	23.83 (5.72)	21.55 (3.95)	31.36 (11.25)	<0.001

GA: Gestational Age, MOM: Mother’s Own Milk, CS: Cesarean Section, MEF: Minimal Enteral Feeding, FEF: Full Enteral Feeding, DM: Donor Milk. The unit of measurement of time is the number of days, the unit of measurement of weight is grams.

**Table 3:** Comparison between the two populations of VLBWs that received and that did not receive Mothers’ Own Milk during hospitalization in NICU, both exclusive and mixed.

	NO	YES	*p*
n°	29	66	
GA (mean (SD))	29.28 (2.95)	28.23 (2.57)	0.084
Weight (mean (SD))	1169.24 (291.62)	1059.64 (281.78)	0.087
Sing_Twins = S (%)	14 (50.00)	44 (66.67)	0.198
CS (%)	24 (82.76)	56 (86.15)	0.910
Maternal Age (mean (SD))	32.00 (7.19)	32.83 (5.68)	0.553
Start MOM (mean (SD))	3.00 (2.83)	4.07 (1.84)	0.429
Length of stay (mean (SD))	57.34 (27.31)	64.59 (25.00)	0.213
Average daily weight increment (mean (SD))	23.38 (4.44)	24.43 (8.26)	0.527
Start MEF (mean (SD))	1.90 (1.18)	1.72 (0.98)	0.457
FEF (mean (SD))	14.88 (7.30)	19.53 (15.73)	0.162

GA: Gestational Age, MOM: Mother’s Own Milk, CS: Cesarean Section, MEF: Minimal Enteral Feeding, FEF: Full Enteral Feeding.

**Table 4:** (**a**) Comparison between the VLBWs discharged with Formulated Milk vs. VLBWs discharged with Mothers’ Own Milk vs. WLBWs discharged with Formulated Milk + Mothers’ Own Milk. (**b**) Comparison between the VLBWs discharged with Formulated Milk and VLBWs discharged with Mothers’ Own Milk (Exclusive MOM + Mixed MOM).

(**a**)				
	**FM**	**MOM**	**MOM + FM**	***p***
GA (mean (SD))	29.12 (2.75)	27.97 (2.43)	27.82 (3.19)	0.101
Weight (mean (SD))	1142.73 (280.65)	1032.29 (284.47)	1042.27 (313.99)	0.182
Single (%)	29 (58.00)	23 (65.71)	8 (72.73)	0.584
CS (%)	42 (82.35)	29 (85.29)	11 (100.00)	0.323
Maternal Age (mean (SD))	31.50 (6.43)	34.68 (5.54)	31.36 (4.90)	0.048
Start MOM (mean (SD))	4.92 (2.54)	3.67 (1.38)	3.50 (0.76)	0.032
Length of stay (mean (SD))	61.28 (28.82)	61.18 (20.34)	69.70 (27.37)	0.622
Average daily weight increment (mean (SD))	24.43 (8.45)	23.52 (5.20)	23.64 (6.94)	0.845
Start MEF (mean (SD))	1.82 (0.93)	1.79 (1.27)	1.55 (0.52)	0.719
FEF (mean (SD))	17.80 (13.97)	17.10 (10.07)	25.45 (22.88)	0.225
(**b**)				
	**FM**	**MOM**	***p***	
GA (mean (SD))	29.12 (2.75)	27.93 (2.59)	0.032	
Weight (mean (SD))	1142.73 (280.65)	1034.67 (288.22)	0.065	
Single (%)	29 (58.00)	31 (67.39)	0.460	
CS (%)	42 (82.35)	40 (88.89)	0.538	
Maternal Age (mean (SD))	31.50 (6.43)	33.87 (5.53)	0.059	
Start MOM (mean (SD))	4.92 (2.54)	3.63 (1.28)	0.009	
Length of stay (mean (SD))	61.28 (28.82)	63.11 (22.08)	0.733	
Average daily weight increment (mean (SD))	24.43 (8.45)	23.55 (5.56)	0.562	
Start MEF (mean (SD))	1.82 (0.93)	1.73 (1.14)	0.670	
FEF (mean (SD))	17.80 (13.97)	19.34 (14.78)	0.620	

GA: Gestational Age, MOM: Mothers’ Own Milk, CS: Cesarean Section, MEF: Minimal Enteral Feeding, FEF: Full Enteral Feeding, FM: Formulated Milk.

## Data Availability

Not applicable.

## Abbreviations

MOM	Mother’s Own Milk
EMOM	Exclusive Mothers’ Own Milk
MMOM	Mixed Mother’s Own Milk
NMOM	No Mother’s Own Milk
HM	Human Milk
DM	Donor Milk
FM	Formula Milk
HMB	Human Milk Bank
BF	Breastfeeding
EBF	Exclusive Breastfeeding
EF	Enteral Feeding
MEF	Minimal Enteral Feeding
FEF	Full Enteral Feeding
VLBW	Very Low Birth Weight
ELBW	Extremely Low Birth Weight
NEC	Necrotizing Enterocolitis
